# Syndecan-1, endocan and non-culprit coronary plaque composition following non-ST elevation myocardial infarction

**DOI:** 10.1016/j.ijcha.2025.101865

**Published:** 2026-01-06

**Authors:** Naomi E. Wattchow, Thalia Salagaras, Mau T. Nguyen, Lauren Y.J. Sandeman, Gemma A. Figtree, Giuseppe Di Giovanni, Dennis T.L. Wong, Stephen J. Nicholls, Christina A. Bursill, Peter J. Psaltis

**Affiliations:** a Vascular Research Centre, Lifelong Health Theme, South Australian Health and Medical Research Institute (SAHMRI), Adelaide, Australia; b Faculty of Health and Medical Sciences, University of Adelaide, Adelaide, Australia; c College of Medicine and Public Health, Flinders University, Adelaide, Australia; d Department of Cardiology, Central Adelaide Local Health Network, Adelaide, Australia; e Faculty of Medicine and Health, University of Sydney, Sydney, Australia; f Kolling Institute of Medical Research, Sydney, Australia; g Victorian Heart Institute, Monash University, Clayton, Australia

**Keywords:** Acute coronary syndrome, Biomarker, Coronary artery disease, Endocan, Glycocalyx, Plaque rupture, Syndecan-1

## Abstract

**Background:**

Syndecan-1 and endocan are biomarkers of endothelial damage, which associate with worse outcomes after myocardial infarction (MI). As it is unclear how this is mediated, we investigated how they associate with the composition of residual, non-culprit coronary atherosclerotic plaques following acute MI.

**Methods:**

This *post hoc* analysis of the COCOMO-ACS trial used serum samples from forty-five patients with non-ST elevation MI who underwent blood collection and optical coherence tomography (OCT) imaging of non-culprit, lipid-rich coronary plaques at baseline and after a median of 17.8 months. Serum syndecan-1 and endocan concentrations at both time-points were measured by ELISA. Relationships between these biomarkers and OCT parameters of rupture-prone plaque were examined.

**Results:**

Serum levels of syndecan-1 (median 161.0 ng/mL at baseline vs 93.5 ng/mL at follow-up, P < 0.0001) and endocan (225.7 pg/mL vs 191.2 pg/mL, P = 0.003) both decreased from time of MI to follow-up, with strong correlation between their changes (R^2^ = 0.64, P < 0.0001). Only syndecan-1 showed a weak negative correlation with minimum fibrous cap thickness at baseline (R^2^ = 0.10, P = 0.03) and a weak positive correlation with maximum lipid arc at follow-up (R^2^ = 0.14, P = 0.01). While syndecan-1 and endocan showed no relationship with plasma lipid concentrations, there were weak associations between follow-up syndecan-1 and interleukin-1-beta (R^2^ = 0.21, P = 0.001), and follow-up endocan and interleukin-6 (R^2^ = 0.15, P = 0.008).

**Conclusions:**

Although serum syndecan-1 and endocan levels decreased in peripheral blood over time post-MI on guideline-directed therapy, this study identified only modest relationships between syndecan-1 (and not endocan) and OCT compositional characteristics of lipid-rich, rupture-prone plaque.

## 1 Introduction

Atherothrombosis leading to acute myocardial infarction (MI) usually occurs because of one of two distinct processes of plaque destabilisation: rupture or erosion [1]. Coronary plaque rupture is predisposed to by thin-cap fibroatheromas (TCFAs) that have a large lipid core and thin fibrous cap with reduced collagen content. Long-term observational studies of atherosclerosis have shown that the presence of such vulnerable plaque features confers increased risk of future cardiac death and MI [2,3]. Mechanisms leading to rupture include a complex interaction of plaque inflammation, necrosis, increased degradation of extracellular matrix and neovascularisation [1,4]. In contrast, plaque erosion occurs when there is loss of integrity of the basement membrane and desquamation of the endothelial cells overlying an intact fibrous cap, with subsequent formation of neutrophil extracellular traps and thrombosis [1,4]. Plaques that erode typically do not have a large lipid core and are mostly either fibrous or thick-cap fibroatheromas with minimum fibrous cap thickness (FCT) ≥ 65 μm [5]. Advances in intracoronary imaging, such as optical coherence tomography (OCT), have revealed that with contemporary treatment of atherosclerotic risk factors (e.g., statins), erosion now accounts for about a third of all acute MI and up to two thirds of non-ST elevation MI (NSTEMI) [1].

Endothelial cell dysfunction and damage are central to the formation of all atherosclerotic plaques, although contribute differently to rupture and erosion [1]. Biomarkers of endothelial injury have therefore been proposed to help identify patients at increased risk of atherosclerotic events. Among these, syndecan-1 is a proteoglycan which forms part of the endothelial glycocalyx [6], the array of highly negatively charged, membrane-bound glycoconjugates on the luminal surface of vascular endothelial cells [7,8]. Syndecan-1 binds and facilitates the interaction of pro-inflammatory cytokines with monocyte-derived macrophages and endothelial cells [9], increases with shear stress and is associated with the activation of endothelial nitric oxide synthase [10]. A murine study showed heightened syndecan-1 expression in the atherosclerotic plaques and serum of *Apoe*^-/-^ mice in association with features of plaque vulnerability, such as increased accumulation of macrophages and foam cells and reduced plaque content of collagen and smooth muscle cells [11]. Endocan, also known as endothelial-specific molecule 1 (ESM1), is a smaller, extracellular soluble proteoglycan expressed and secreted by endothelial cells and promotes leukocyte adhesion and proliferation of vascular smooth muscle cells [12,13]. Elevation of circulating levels of each biomarker is thought to reflect damage to the endothelium or shedding of its glycocalyx [10,14] and may have prognostic implications in sepsis, cancer and coronary artery disease, including poorer outcomes after acute MI [15,16]. To explore how this may be mediated, this study examined how serum concentrations of syndecan-1 and endocan change over time post-MI and associate with OCT-based measurements of the composition of residual lipid-rich, rupture-prone coronary plaques.

## 2 Methods

In this *post hoc* exploratory analysis, imaging data and blood samples were used from the COCOMO-ACS study (ANZCTR trial registration number: ACTRN12618000809235) [17,18]. This multicentre, randomised, double-blind, placebo-controlled trial was performed in patients with NSTEMI to evaluate the effect of the anti-inflammatory drug, colchicine, on non-culprit plaque using serial OCT. The study was conducted in accordance with the Declaration of Helsinki (as revised in 2013) and was approved by the Royal Adelaide Hospital Human Research Ethics Committee under the National Mutual acceptance system with local Research Governance Officer approval at each site (HREC/17/RAH/366; Central Adelaide Local Health Network Reference Number R20170904). Informed consent was obtained from all individual participants.

Patients with NSTEMI underwent coronary angiography within 72 h of admission with management of their culprit coronary lesion as clinically indicated. Eligible patients needed to have a non-obstructive lesion in a non-culprit artery of 20-50 % stenosis, which was imaged by OCT with the Dragonfly^TM^ OPTIS^TM^ Imaging Catheter (Abbott Medical/Lightlab Imaging, Inc., Westford, MA) at baseline and 12–18 months later. This lesion needed to contain a lipidic plaque with at least one frame with FCT ≤ 120 µm and total lipid arc >90°. 64 participants were then randomised 1:1 to receive colchicine 0.5 mg/d or placebo in addition to standard guideline recommended pharmacotherapy.

Each participant had OCT imaging of only one non-culprit coronary artery. We have previously published our core laboratory’s methods, reproducibility and accuracy of OCT image analysis to identify compositional characteristics that associate with increased risk of plaque rupture. This includes high interobserver and intraobserver reliability for FCT (κ > 0.92 for both) and lipid arc (κ > 0.98 for both) [19]. Core laboratory personnel analysed OCT cross-sectional images at 0.2 mm spacing, blinded to treatment allocation and imaging sequence. The segment selected for analysis was defined by proximal and distal side branches. Analysts used manual planimetry to measure the minimum FCT (determined as the thinnest point selected for measurement) and lipid arc for each evaluable image [19,20]. Each image was also scored for presence of macrophages, microchannels, plaque rupture and TCFA, which was defined as a plaque with lipid arc >90° and overlying thin fibrous cap (<65 µm). To measure the macrophage composition for each plaque, each frame received a score from 0 to 4 based on total arc of macrophages (e.g., 1 = 1–90°), which was then summed across all frames within the plaque, divided by the total number of frames and multiplied by 5 to give the macrophage composition per mm of vessel. The number of microchannels per mm was calculated similarly. All measurements made by an analyst were subsequently reviewed by another core laboratory member and a medical reviewer.

Blood was taken, processed and cryopreserved at baseline and follow-up angiography. Clinical testing was performed by the local laboratory for biochemistry, estimated glomerular filtration rate (eGFR) and lipids (low-density lipoprotein-cholesterol [LDL-C], high-density lipoprotein-cholesterol [HDL-C], triglycerides). Sandwich enzyme-linked immunosorbent assay (ELISA) kits were used to measure serum concentrations of syndecan-1 (human syndecan-1 CD138 Abcam ab46506) and endocan (human ESM1 endocan Abcam ab213776) in single batch analysis, along with interleukin-6 (IL-6) (R&D Systems Human IL-6 Quantikine RDSD6050), interleukin-1-beta (IL-1β) (R&D Systems Human IL-1β Quantikine RDSHSB00D) and high-sensitivity C-reactive protein (hs-CRP) (Abacus Demeditec hsCRP 96T DMDE740011).

### 2.1 Sample size and statistical analysis

The parent COCOMO-ACS study was powered to detect a 50 % difference between colchicine and placebo for the percentage increase in minimum FCT, giving an overall target sample size of N = 52 [17,18]. Allowing for loss to follow-up, 64 participants were randomised. 57 completed the protocol with analysable OCT imaging of non-culprit vessels at both time-points. Of these, 45 had blood samples available from baseline and follow-up visits that could be used for this exploratory analysis.

GraphPad prism V10, R Studio (Version 2023.06.02) and Stata 18.5 (College Station, Texas, US) were used for statistical analysis. Results were tested for normality of distribution by the Shapiro-Wilk test and are summarised as median with interquartile range (IQR) for non-normally distributed data. Groups were compared by paired or unpaired t-tests (or their non-parametric versions) and relationships between different parameters further analysed by linear regression. Two-tailed P-values < 0.05 were deemed significant.

## 3 Results

Characteristics of the 45 participants in this analysis are summarised in Table 1. 23 were allocated placebo and 22 colchicine, with median follow-up 17.8 (IQR 16.6–18.2) months. 41 (91.1 %) finished the study on high-intensity statins, with plasma LDL-C reducing from 2.8 (IQR 2.1–3.6) mmol/L at baseline to 1.5 (IQR 1.1–2.1) mmol/L at follow-up in the overall cohort (P < 0.0001). The parent study found no significant overall effect for colchicine compared to placebo on changes in the minimum FCT or maximum lipid arc from baseline to follow-up in the imaged vessels [17,18]. To examine relationships between syndecan-1, endocan and plaque, we therefore combined results of both the placebo and colchicine groups. Table 2 shows the changes in OCT measures of plaque composition. The minimum FCT in imaged segments increased by a median of 52.5 % and maximum lipid arc decreased by 18.2 % (P < 0.0001 for both). The presence of non-culprit TCFAs decreased from 66.7 % to 0 % (P < 0.0001), indicating that most participants displayed stabilisation of their imaged, non-culprit plaques.

**Table 1:** Participant characteristics.

**Variable**	**Result**
Age, years	61 (57–71)
Males	40 (88.9 %)
Follow-up, months	17.8 (16.6–18.2)
Hypertension	22 (48.9 %)
Diabetes	8 (17.8 %)
Previous MI	3 (6.7 %)
Smoking (current)	10 (22.2 %)
Prior statin use	13 (28.9 %)
High-intensity statin (end of study)	41 (91.1 %)
BMI, kg/m^2^	29.5 (26.1–33.8)
eGFR, mL/min/1.73 m^2^ (baseline)	92 (75–99)
LDL-C, mmol/L (baseline)	2.8 (2.1–3.6)
LDL-C, mmol/L (follow-up)	1.5 (1.1–2.1)
hs-CRP, mg/L (baseline)	3.5 (1.7–14.9)
hs-CRP, mg/L (follow-up)	0.8 (0.5–1.7)
IL-1β, pg/mL (baseline)	0.10 (0.02–0.21)
IL-1β, pg/mL (follow-up)	0.05 (0.02–0.10)
IL-6, pg/mL (baseline)	2.7 (1.8–11.8)
IL-6, pg/mL (follow-up)	2.4 (1.3–3.9)

Categorical data are presented as N (%) and continuous data as median (IQR). N = 45. BMI, body mass index; eGFR, estimated glomerular filtration rate; HDL-c, high-density lipoprotein cholesterol; hs-CRP, high-sensitivity C-reactive protein; IL-1β, interleukin-1β; IL-6, interleukin-6; LDL-c, low-density lipoprotein cholesterol; MI, myocardial infarction.

**Table 2:** Optical coherence tomography results for imaged arteries.

**OCT measures**	**Baseline**	**Follow-up**	**Absolute change**	**Percentage change**	**P value**
FCT_min_ (μm)	61.0 (57.5–64.5)	93.0 (82.0–110.0)	+36.1 (19.7–72.9)	+52.5 % (42.6 %–70.5 %)	**<0.0001**
Lipid arc_max_ (°)	187.0 (157.0–245.0)	153.0 (115.5–196.5)	−43.0 (−72.5 to −17.0)	−18.2 % (−26.4 to −19.7 %)	**<0.0001**
Rupture present	6 (13.3 %)	9 (20.0 %)	+3	+50 %	0.51
TCFA present	30 (66.7 %)	0 (0.0 %)	−30	−100 %	**<0.0001**
Macrophage composition (per mm)	3.5 (2.8–4.3)	3.2 (2.1–4.0)	−0.5 (−1.1 to 0.3)	−0.1 % (−0.3 to −0.1 %)	**0.02**
Microchannels (per mm)	0.4 (0.0–1.3)	0.4 (0.0–1.1)	0.0 (−0.6 to 0.1)	−0.1 % (−0.2 to 0.0 %)	0.12

Results for optical coherence tomography (OCT) measures of non-culprit plaque composition at baseline and follow-up and their percentage change over time. Categorical data are presented as N (%) and continuous data as median (IQR). N = 45. FCT_min_, minimum fibrous cap thickness; Lipid arc_max,_ maximum lipid arc; TCFA, thin-cap fibroatheroma.

**Fig. 1:**
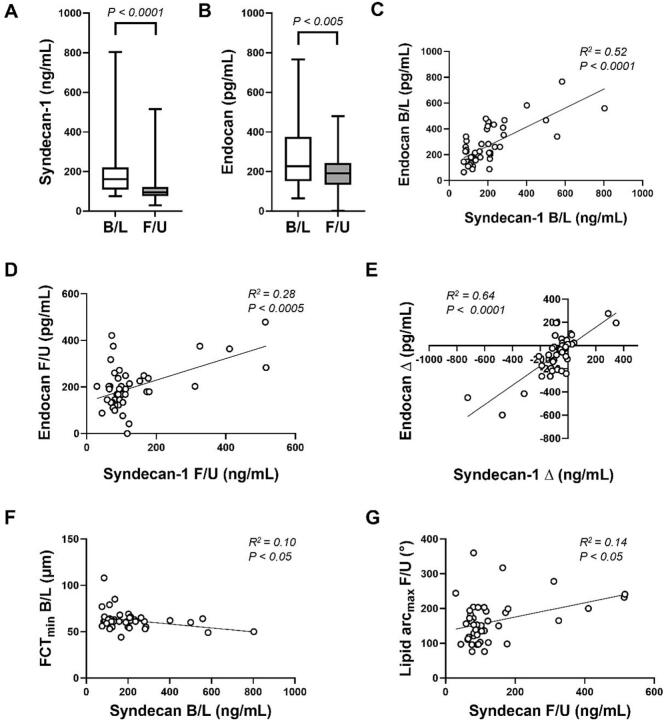
**Syndecan-1 and endocan levels and their relationship with each other and plaque characteristics.** Graphs show median (and IQR) concentrations in serum for (A) syndecan-1 and (B) endocan at baseline (B/L) and follow-up (F/U), and linear regression analysis between both biomarkers at (C) baseline, (D) follow-up, and (E) their changes (Δ) over time (E). Relationship between (F) syndecan-1 with minimum fibrous cap thickness (FCT_min_) at baseline and with (G) lipid arc max (lipid arc_max_) at follow-up by linear regression analysis and Pearson’s correlation. N = 45. Statistical comparisons performed by Wilcoxon signed rank test in (A) and (B).

**Fig. 2:**
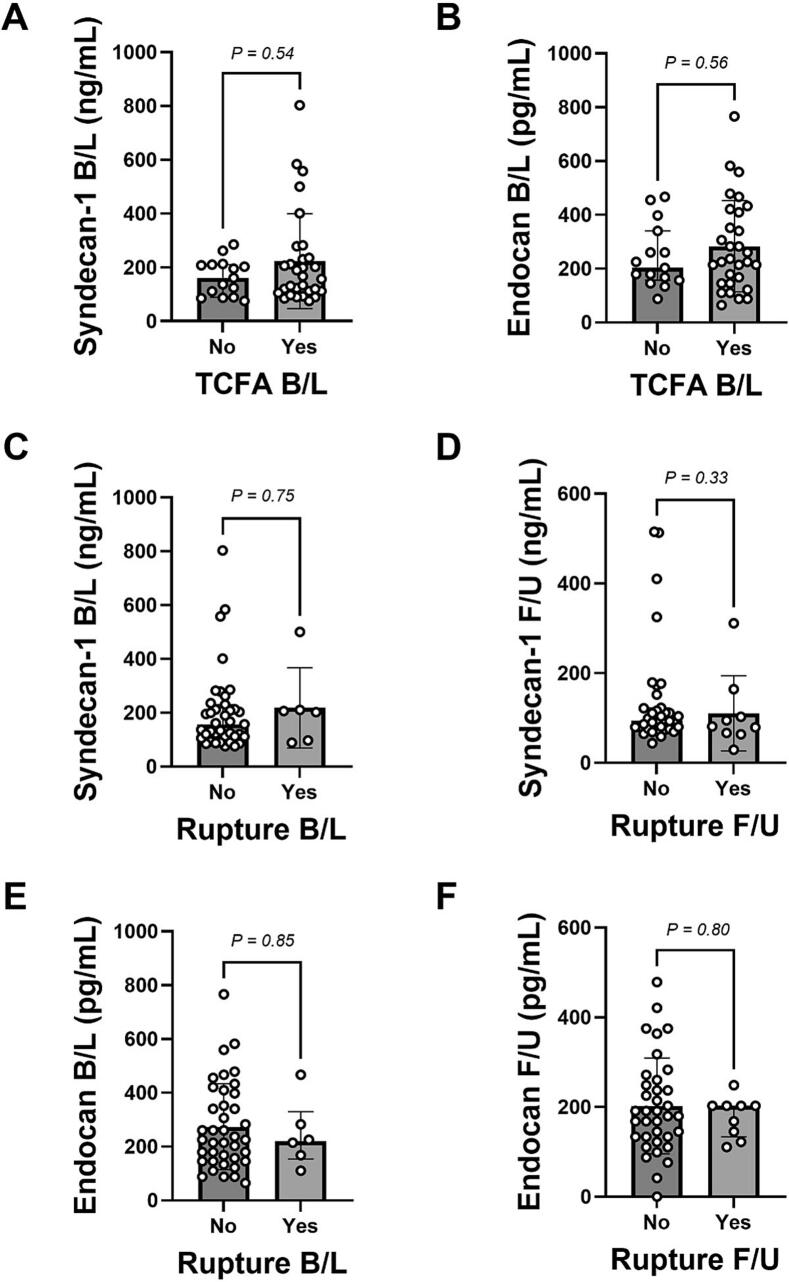
**Syndecan-1 and endocan levels according to presence of TCFA and plaque rupture.** Graphs show syndecan-1 and endocan levels according to the presence of thin-cap fibroatheromas (TCFAs) in the imaged non-culprit artery at baseline (B/L) (i.e., soon after the index MI), or plaque rupture at baseline or follow-up (F/U). Data are summarised as median (IQR). Statistical analysis performed by Mann-Whitney tests. N = 45.

There were significant decreases in serum levels from baseline to follow-up of syndecan-1 (161.0 ng/mL [IQR 108.4–221.6 ng/mL] versus 93.5 ng/mL [IQR 75.8–122.2 ng/mL]; median change −26.7 %; P < 0.0001) and endocan (225.7 pg/mL [IQR 150.9–375.2 pg/mL] versus 191.2 pg/mL [IQR 133.7–242.9 pg/mL]; median change −10.2 %; P = 0.003) (Fig. 1A, B3), with no differences between patients allocated to colchicine or placebo (Supplemental Fig. 1). These two markers correlated positively with each other at each time-point, as well as for their respective changes over time (R^2^ = 0.64, P < 0.001) (Fig. 1C-E). We found no differences for syndecan-1 or endocan levels based on the presence or absence of non-culprit TCFAs around the time of MI, or non-culprit plaque rupture at either time-point (Fig. 2).

**Fig. 3:**
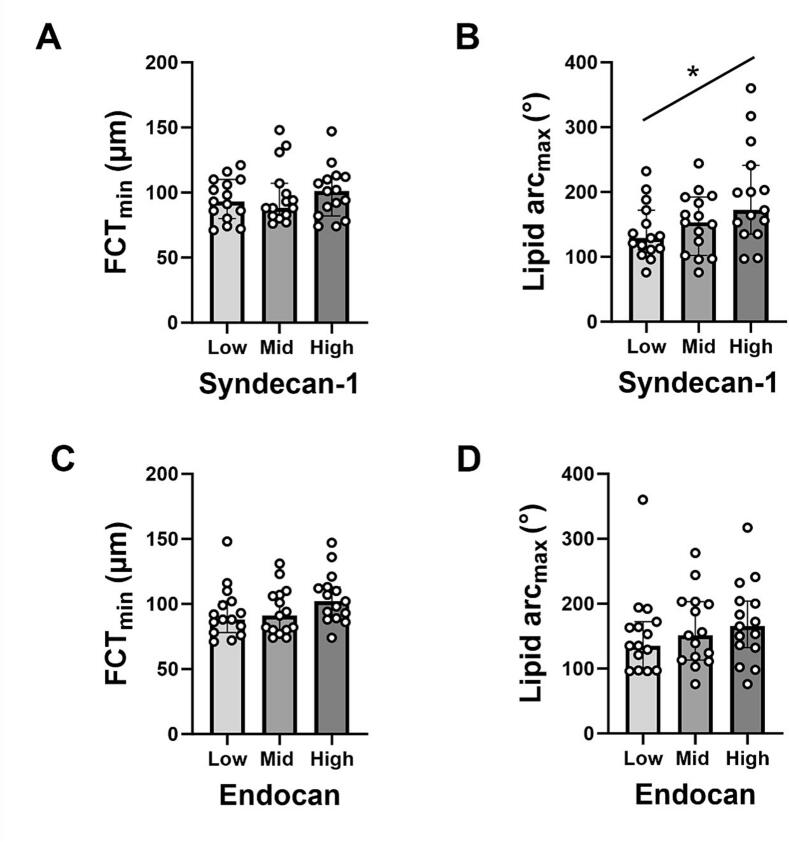
**Plaque characteristics at follow-up according to tertiles of syndecan-1 and endocan.** Graphs show the results for minimum fibrous cap thickness (FCT_min_) and maximum lipid arc (lipid arc_max_) according to tertiles for serum levels of syndecan-1 and endocan at follow-up. Data are summarised as median (IQR). Statistical analysis performed by Kruskal-Wallis tests with all P values not significant. * P < 0.05 for linear trend analysis. N = 15 for each tertile group.

Generalised linear mixed models, adjusted for age, sex, LDL-C and study visit, showed no significant relationships between syndecan-1 or endocan and minimum FCT or maximum lipid arc. However, using generalised linear regression we identified an association between syndecan-1 and minimum FCT at baseline visit on unadjusted analysis, and after adjustment for age, sex and LDL (both P = 0.03), with weak negative correlation (R^2^ = 0.10, P = 0.03) (Table 3**,** Fig. 1F**)**. Meanwhile, at follow-up visit there was a signal toward a weak positive correlation between syndecan-1 and maximum lipid arc (R^2^ = 0.14, P = 0.01) (Table 3**,** Fig. 1G), although this did not reach significance by generalised linear regression (unadjusted P = 0.06; adjusted P = 0.10). Neither biomarker showed significant associations with the other OCT parameters studied (Table 3), or with minimum FCT or maximum lipid arc by tertile-based comparisons at follow-up, although there was a trend towards increased maximum lipid arc with the highest syndecan-1 tertile (Fig. 3).

**Table 3:** Relationship between biomarkers and OCT parameters at different time-points.

Results are summarised as R^2^ values from linear regression analysis using Pearson’s correlation coefficient. N = 45. Statistically significant results are indicated in bold font and designated as * P < 0.05, † P < 0.01, ‡ P < 0.001, § P < 0.0001. Other results were not statistically significant. FCT_min_, minimum fibrous cap thickness; HDL-c, high-density lipoprotein cholesterol; hs-CRP, high-sensitivity C-reactive protein; IL-1β, interleukin-1-beta; IL-6, interleukin-6; Lipid arc_max_, maximum lipid arc; LDL-c, low-density lipoprotein cholesterol; TG, triglycerides; Trop T, troponin.

With respect to other biomarkers of atherosclerotic risk, we identified significant but modest correlations between baseline syndecan-1 and triglycerides, follow-up syndecan-1 and serum IL-1β, and follow-up endocan and IL-6, but not with hs-CRP or lipoprotein cholesterol levels (Table 3).

## 4 Discussion

Although syndecan-1 and endocan are biomarkers of interest for measuring endothelial injury [6,8] and ischaemic risk [15,16], it has been unclear to what extent they reflect the natural history of coronary atherosclerosis. The most definitive results from this *post hoc* analysis of the COCOMO-ACS study are that serum syndecan-1 and endocan levels decreased over time in the context of guideline-recommended therapy post-MI and associated closely with each other, with no treatment effect from colchicine. However, among different OCT measures of non-culprit plaque composition, we only identified a weak relationship between syndecan-1 and minimum FCT in the early post-MI setting, and potentially between syndecan-1 and maximum lipid arc at follow-up.

Prior studies have shown that syndecan-1 and endocan levels are increased after acute coronary syndrome [16,21], and have suggested that both associate with recurrent ischaemic events [15,16,22]. For example, in a cohort of 206 patients with STEMI, higher plasma syndecan-1 levels associated with an increased rate of major adverse cardiac events and mortality at six months and showed higher discriminative power than troponin and creatine kinase [15]. Furthermore, in a large study of patients with stable coronary artery disease, it was found to associate with increased cardiovascular events over follow-up of 24 months [22]. Similarly, endocan has been shown to discriminate patients with STEMI and higher anatomical complexity of coronary disease [16], and those who are more likely to develop in-stent re-stenosis [23]. To our knowledge, the current study is the first to serially track syndecan-1 and endocan levels in parallel with coronary plaque imaging over an extended follow-up period post-MI. Although both markers significantly decreased, the *post hoc* design of our analysis precludes the ability to interpret whether this occurred as part of the natural recovery process following acute MI or whether it was a product of effective secondary prevention therapy, which included a high rate of statin use and the lowering of LDL-cholesterol and inflammatory biomarkers (hs-CRP, IL-6 and IL-1β). Biomarker kinetics could certainly have been influenced by differences in follow-up duration, treatment intensity and risk factor control between participants, which were well matched between the placebo and colchicine groups of the parent study [18,25].

Previously, Nemoto *et al*. reported that in patients with stable coronary artery disease, those with serum syndecan-1 levels below the study’s median value of 99.0 ng/mL had higher prevalence of lipid-rich plaque and TCFAs [24]. In contrast, our analysis of baseline samples in COCOMO-ACS suggested a weak negative correlation between syndecan-1 and minimum FCT in non-culprit plaques, meaning that higher levels associated with thinner fibrous cap soon after NSTEMI presentation. However, we could not reproduce a relationship between syndecan-1 (or endocan) and the presence of non-culprit TCFAs or ruptured plaques. Meanwhile, at best only a tenuous correlation between higher syndecan-1 levels and higher maximum lipid arc was identified at follow-up, with no suggestion that either biomarker associated with other OCT parameters measured here, or their change over time. Based on these findings, syndecan-1 and endocan appear to be poorly representative of lipid-rich TCFAs that carry increased risk of plaque rupture. Instead, as markers of endothelial injury, we hypothesise that they could still associate with plaques that have undergone, or are at risk of undergoing plaque erosion, which is mediated more acutely by endothelial cell dehiscence and loss. However, as no OCT-erosion phenotype was studied here, this hypothesis would need evaluation in future studies.

This *post hoc* exploratory study has several limitations, including a small sample size, lack of comparison against a healthy control group, and the unintended low recruitment of female patients. The ELISA results for syndecan-1 and endocan are laboratory and kit-specific. Neither is a specific biomarker of cardiac endothelium, and blood was sampled peripherally for pragmatic reasons, rather than from coronary sinus sampling which may have provided different measurements more directly reflective of plaque biology. Other studies which have recruited mixed types of patients with acute coronary syndrome have reported lower median biomarker values, whereas we only included those with NSTEMI who had residual atherosclerotic disease. Finally, our analysis was constrained by the specific design of the original COCOMO-ACS study, whereby OCT imaging was performed in only one non-culprit artery for each participant, focusing on non-obstructive, lipid-rich plaques. Our results are therefore not representative of culprit lesions responsible for index MI, non-culprit obstructive plaques nor different types of plaque with non-lipidic composition. They also do not reflect the global burden of plaque throughout the whole coronary and non-coronary vasculature.

In conclusion, this study found significant reductions in serum levels of syndecan-1 and endocan on guideline-recommended therapies post-MI, but at most only weak associations between syndecan-1 and high-risk morphological features in non-culprit, lipid-rich coronary plaque. Overall, it remains unclear whether the reduction in both biomarkers over time was due to post-MI recovery with regression to baseline variability, or the effect of secondary prevention therapies like statins. However, there was no treatment effect between colchicine and placebo.

## Ethical statement

The authors are accountable for all aspects of the work in ensuring that questions related to the accuracy or integrity of any part of the work are appropriately investigated and resolved. The study was conducted in accordance with the Declaration of Helsinki (as revised in 2013). The study was approved by the Royal Adelaide Hospital Human Research Ethics Committee under the National Mutual acceptance system with local Research Governance Officer approval at each site (HREC/17/RAH/366; Central Adelaide Local Health Network Reference Number R20170904) and informed consent was obtained from all individual participants.

## CRediT authorship contribution statement

**Naomi E. Wattchow:** Writing – review & editing, Writing – original draft, Visualization, Software, Methodology, Investigation, Formal analysis, Data curation, Conceptualization. **Thalia Salagaras:** Writing – review & editing, Investigation. **Mau T. Nguyen:** Writing – review & editing, Investigation, Formal analysis. **Lauren Y.J. Sandeman:** Writing – review & editing, Investigation. **Gemma A. Figtree:** Writing – review & editing. **Giuseppe Di Giovanni:** Writing – review & editing, Formal analysis. **Dennis T.L. Wong:** . **Stephen J. Nicholls:** Writing – review & editing. **Christina A. Bursill:** Writing – review & editing. **Peter J. Psaltis:** Writing - original draft, Writing - review & editing, Investigation, Formal Analysis, Data curation, Conceptualization, Funding, Supervision.

## Funding

This investigator-initiated study was funded by project grants from the National Health and Medical Research Council of Australia (NHMRC ID 1127159), National Heart Foundation of Australia (NHF Vanguard Grant ID 10137) and the Sylvia and Charles Viertel Charitable Foundation (VIERCI 2017016). D.T.L.W. has received a Level 1 Future Leader Fellowship from the National Heart Foundation of Australia [FLF 102535]. S.J.N. has received a Principal Research Fellowship from the NHMRC [ID 1111630]. P.J.P. receives a Level 3 Future Leader Fellowships from the National Heart Foundation of Australia [FLF 106656].

## Declaration of competing interest

The authors declare the following financial interests/personal relationships which may be considered as potential competing interests: [G.A.F. has received personal fees from CSL and CPC Clinical Research and is Founding Director/Chief Medical Officer of Prokardia and Chief Scientific Officer of CAD Frontiers. D.T.L.W. has received speaker honoraria from AstraZeneca, Pifzer, Bayer and Boehringer Ingelheim. S.J.N. has received research support from AstraZeneca, Amgen, Anthera, CSL Behring, Cerenis, Eli Lilly, Esperion, Resverlogix, Novartis, InfraReDx and Sanofi-Regeneron and is a consultant for Amgen, Akcea, AstraZeneca, Boehringer Ingelheim, CSL Behring, Daiichi Sankyo, Eli Lilly, Esperion, Kowa, Merck, Takeda, Pfizer, Sanofi-Regeneron, Novo Nordisk, CSL Seqirus and Vaxxinity. P.J.P. has received research support from Amgen and Biotronik, consulting fees from Amgen, CSL Seqirus, Esperion, Eli Lilly, Novartis, Novo Nordisk and Sanofi, and speaker honoraria from Amgen, CSL Seqirus, Novartis, Novo Nordisk and Sanofi and is a non-executive board director of Corcillum Systems].
