# Supplementary material for: Syndecan-1, endocan and non-culprit coronary plaque composition following non-ST elevation myocardial infarction
Source: Int J Cardiol Heart Vasc. 2026 Jan 6;62:101865. doi: 10.1016/j.ijcha.2025.101865 (PMC13153135; doi:10.1016/j.ijcha.2025.101865)
Supplement: Supplementary Data 1 [file mmc1.docx]

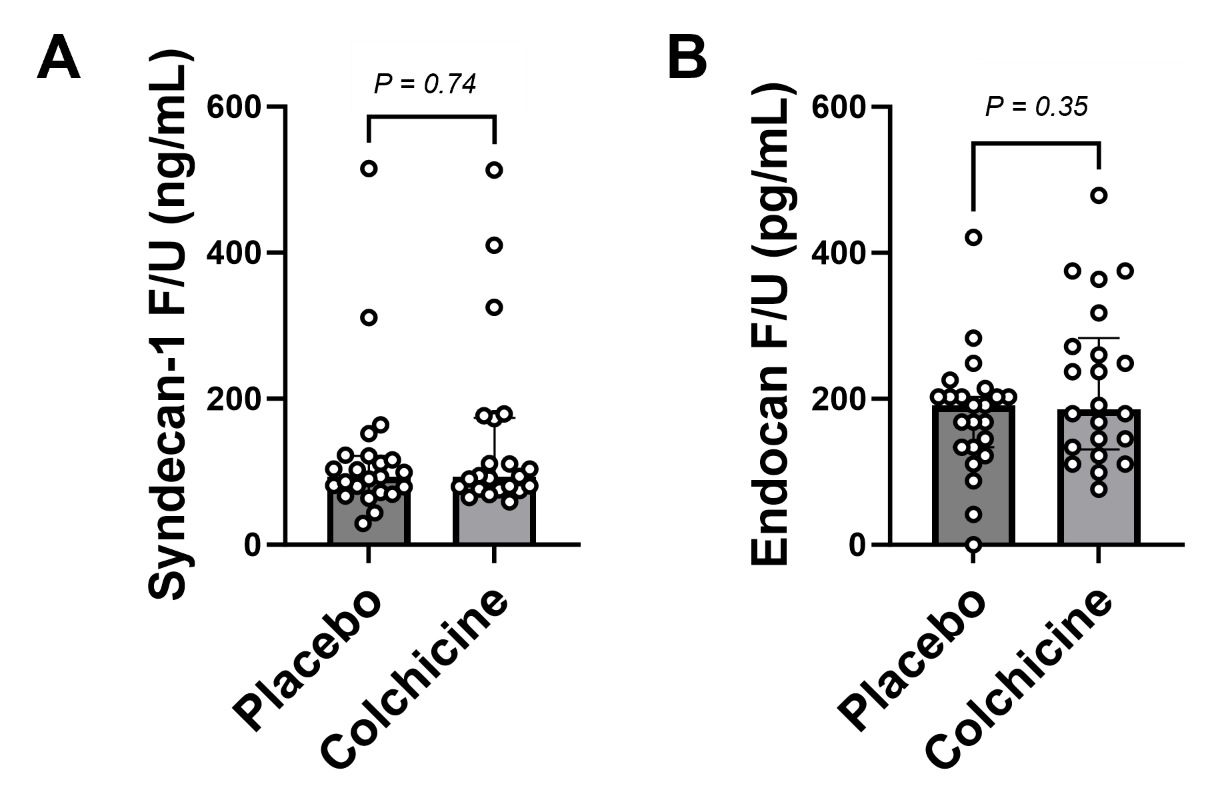


**Supplemental Figure 1. Follow-up syndecan-1 and endocan levels by study group allocation.** Graphs show serum levels for (A) syndecan-1 and (B) endocan between colchicine and placebo groups at follow-up (F/U). Data are summarised as median (IQR). Statistical comparisons performed by Mann-Whitney tests. N=22 for colchicine and N=23 for placebo.
